# The traumatic brain injury mitigation effects of a new viscoelastic add-on liner

**DOI:** 10.1038/s41598-019-39953-1

**Published:** 2019-03-05

**Authors:** Petros Siegkas, David J. Sharp, Mazdak Ghajari

**Affiliations:** 1 Dyson School of Design Engineering, Imperial College London, South Kensington Campus, London, SW7 2AZ UK; 2 The Computational, Cognitive and Clinical Neuroimaging Laboratory, Division of Brain Sciences, Imperial College London, London, W12 0NN UK

## Abstract

Traumatic brain injury (TBI) affects millions of people worldwide with significant personal and social consequences. New materials and methods offer opportunities for improving designs of TBI prevention systems, such as helmets. We combined empirical impact tests and computational modelling to test the effectiveness of new viscoelastic add-on components in decreasing biomechanical forces within the brain during helmeted head impacts. Motorcycle helmets with and without the viscoelastic components were fitted on a head/neck assembly and were tested under oblique impact to replicate realistic accident conditions. Translational and rotational accelerations were measured during the tests. The inclusion of components reduced peak accelerations, with a significant effect for frontal impacts and a marginal effect for side and rear impacts. The head accelerations were then applied on a computational model of TBI to predict strain and strain-rate across the brain. The presence of viscoelastic components in the helmet decreased strain and strain-rate for frontal impacts at low impact speeds. The effect was less pronounced for front impact at high speeds and for side and rear impacts. This work shows the potential of the viscoelastic add-on components as lightweight and cost-effective solutions for enhancing helmet protection and decreasing strain and strain-rate across the brain during head impacts.

## Introduction

Traumatic brain injury (TBI) has vast public health impact with an estimated 5.3 million people living with TBI related disability in the USA and 7.7 million people with TBI related disability in EU^1,2^. Unfortunately, the number of TBI cases is rising worldwide and this rise has been related to the increase in use of motor vehicles^3^. Particularly significant number of road accidents involve motorcycles, in some cases up to 50% e.g. in Malaysia^4^. Head injuries contribute to more than 40% of motorcycle fatalities in Malaysia^5^, 43% in Germany and 53% in USA^4^. Motorcyclists are more exposed to impact. According to a report from Lin and Kraus^6^, motorcycle riders are more than 30 times more likely to die in a traffic crash than car occupants, hence the importance of effective protective equipment and the need for improvements in TBI prevention systems to better mitigate short and long-term effects of head impacts. Safety helmets are designed to prevent TBI by reducing the effects of the forces applied to the head. Helmet design, however, has not been significantly improved in the past decades.

New materials and technologies offer promising opportunities in designing better protective equipment, e.g. improved helmets. Viscoelastic materials have characteristics that hold promise for enhancing head protection equipment^7,8^. The mechanical response of these materials is dependent on the rate of deformation; they become stiffer at high rates of deformation. This property can make these materials absorb more energy when deforming faster under impact. Under lower rates of deformation they remain soft hence improving comfort of helmets during fitting and wearing under normal riding conditions.

**Figure 1:**
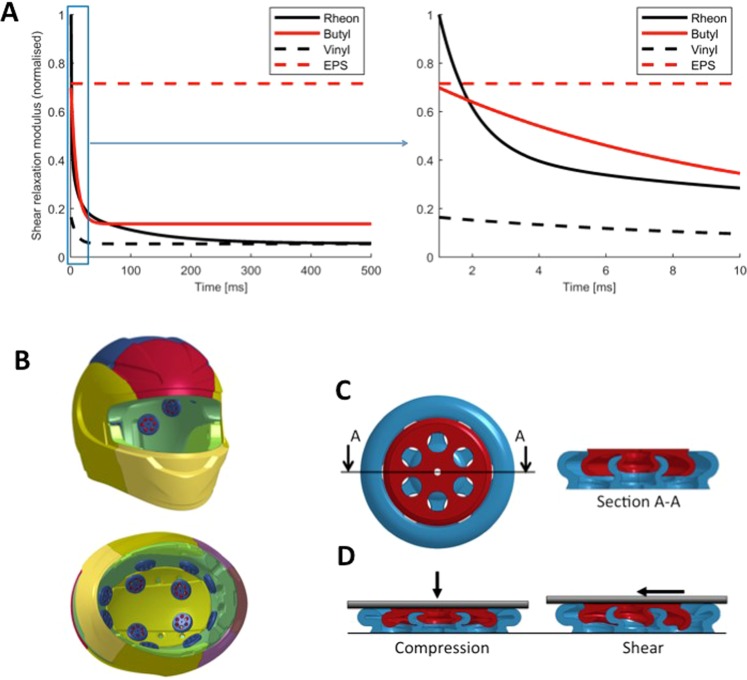
(**A**) Shear relaxation modulus of Rheon compared with two elastomers and the shear modulus of EPS foam (40 g/l). (**B**) The helmet fitted with the viscoelastic components (CAD images published with kind permission by Dainese), (**C**) the components are composed of a small revolver and large revolver, fitted together, (**D**) the component designed to exhibit different deformation responses under compression and shear loading (CAD images published with kind permission by Rheon Labs).

Here we investigate the head protection performance of a new viscoelastic add-on liner that is combined with an existing helmet design. The liner, which weighs only 4% of the helmet mass, is composed of components made of a dilatant viscoelastic material (Fig. 1A) (see the methods section) with a potentially versatile geometry. It can be retrofitted to existing helmets (Fig. 1B,C and the methods section). The shear relaxation of the material, shown in Fig. 1A, indicates the highly viscoelastic nature of this material with a short-term stiffness nearly 15 fold larger than its long-term stiffness. The component is made of two disks fitted together through their central parts. Due to its geometry and the interaction between the two constituent disks, the viscoelastic component has different responses under compressive and shear deformation (Fig. 1D), providing an opportunity to optimise its design for tangential and normal components of the force in oblique impacts. The viscoelastic component is more compliant in shear than in compression. Enhanced shear compliance could potentially reduce the rotational acceleration of the head produced by the shear force.

The majority of studies on new helmet designs have used head translational acceleration as the indicator of traumatic brain injury^9–12^. However, the exclusive use of translational accelerations as a helmet design criterion is arguably insufficient. Studies have suggested including rotational effects of impacts, e.g. head rotational acceleration, in new brain injury metrics improves their ability to predict TBI^13–15^. However both translational and rotational accelerations are based on the global kinematics of the head, ignoring its complex structure. Cadaver experiments using x-ray videography and neutral density targets implanted in the brain to measure brain deformation^16^ and finite element modelling of brain injuries in sporting collisions^17^ have shown that in order to improve TBI prediction, we should predict the brain response to head accelerations rather than restricting the focus to accelerations only. Specifically, strain and strain rate within the brain have been shown to be promising predictors of TBI^17^. Hence a potentially better approach to testing the protection of helmets is using finite element models of TBI, which include the anatomy and biomechanical properties of different tissues.

**Figure 2:**
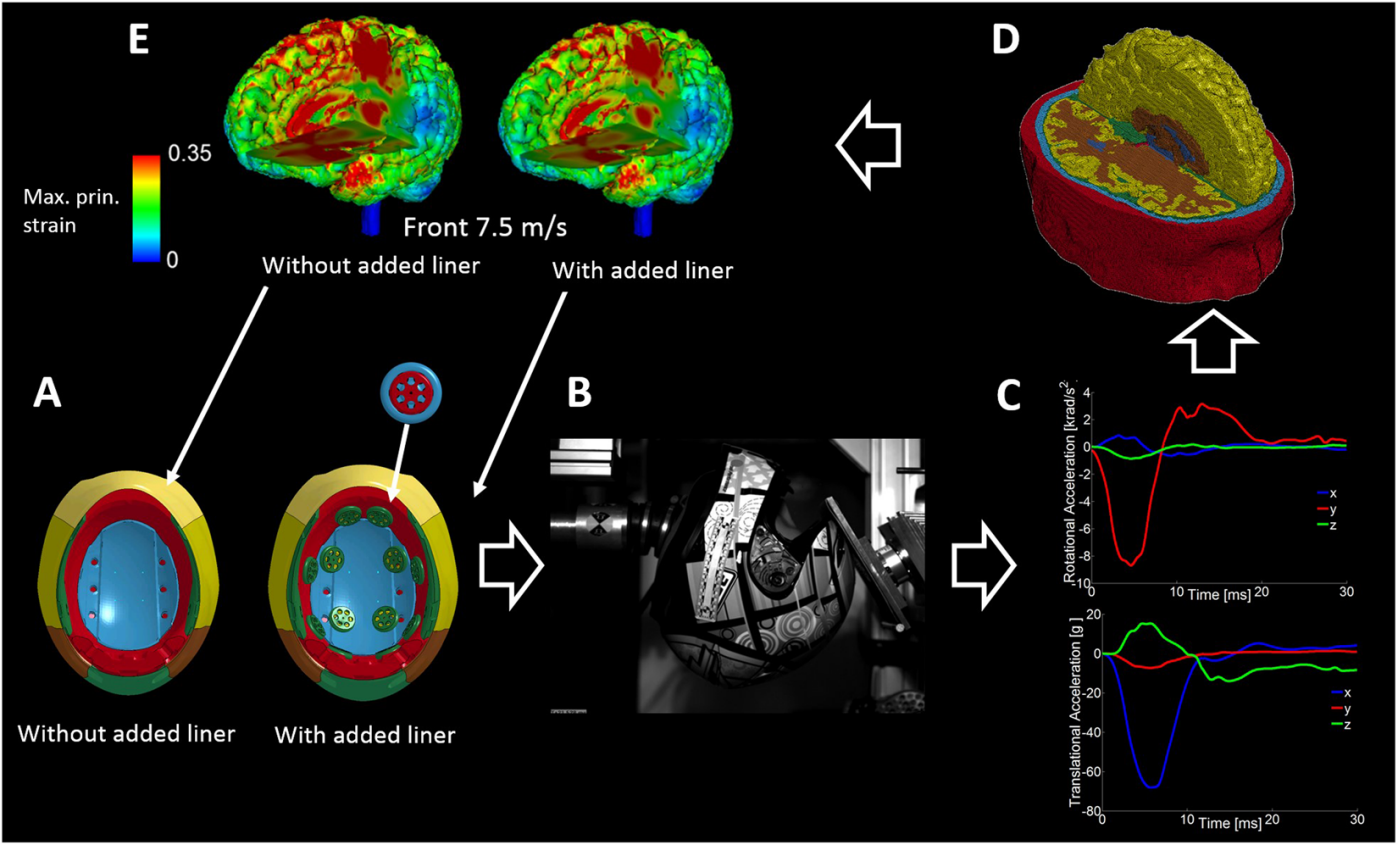
Methods (**A**) The viscoelastic components were fitted on the interior surface of the helmet liner. (**B**) Helmets with and without the added components were tested using a drop tower apparatus fitted with an oblique anvil. The helmets were fitted on an instrumented Hybrid III head-form, attached to a Hybrid III neck. The neck was connected to a suspended pendulum structure designed to mimic inertial effects of the body. (**C**) Translational and rotational acceleration traces on the XYZ axes, obtained via accelerometers embedded within the Hybrid III head-form. (**D**) Our computational model of TBI was loaded with the translational and rotational accelerations. (**E**) Strains and strain rates within the brain were mapped and compared.

In this study, we test whether retrofitting the viscoelastic components to the helmet liner would improve the helmet protection. To this end, we compared the response of the helmet with and without the add-on liner, with control experiments being the experiments on the helmets without the add-on liner. We combined empirical and computational approaches to test the protection of helmets with the viscoelastic components. An oblique impact rig was used (Fig. 2B), which was fitted with a Hybrid III head/neck assembly attached to a pendulum and an oblique monorail impactor. The impact sites were front, side and rear. We performed impacts at 4.35 m/s representing a low speed impact equivalent to falling from a 1 m height and at 7.5 m/s, prescribed by the ECE22.05 motorcycle helmet standard^11^, and we recorded translational and rotational accelerations of the headform (Fig. 2C). The accelerations were then applied on an FE model of TBI that included the detailed anatomy of the human head (Fig. 2D). This model has been used previously to predict the location of pathology seen in a neurodegenerative disease, chronic traumatic encephalopathy^18^. Here we used this model to predict strains and strain rates across the brain (Fig. 2E), which have been linked to cell death and neurodegeneration after TBI^19–21^. This will allow us to better examine the protection offered by using viscoelastic add-on components in an existing helmet design.

## Results

### The effect of viscoelastic components on peak head accelerations

**Figure 3:**
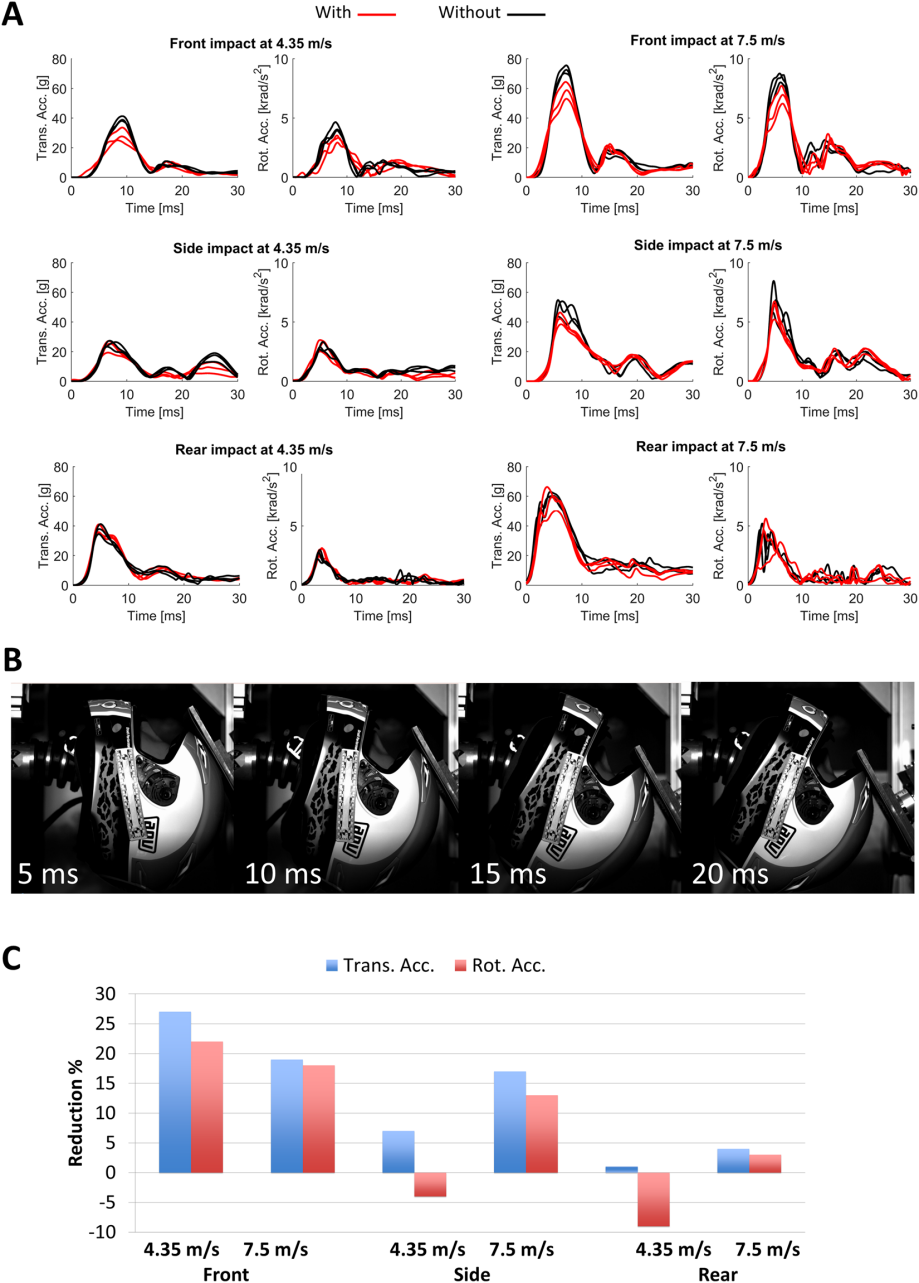
(**A**) Head translational (left) and rotational (right) accelerations for impacts with (red line) and without (black line) the viscoelastic components. Tests were repeated three times, each time on a different helmet; (**B**) Snapshots of the frontal impact at 7.5 m/s; (**C**) Improvement in average peak accelerations (rotational and translational) for helmets with viscoelastic components.

Figure 3(A) shows head resultant accelerations for all impacts. Both translational and rotational accelerations reach a peak value in the first 10–15 ms of the impact, which is associated with the contact between the anvil and helmet (Fig. 3B). The contact force rotates the helmet and headform and bends the neck. The rotation stops at around 15 ms and accelerations approach zero, which is due to the balance between the anvil force and the neck force and moment exerted on the head. As the anvil force decreases further, the neck force and moment produce another peak in head accelerations, but this peak is smaller than the first peak, which is directly caused by the impact.

Peak translational and rotational accelerations of the head have been suggested as indicators of traumatic brain injury^17^. We investigated the effects of including the viscoelastic components on these quantities (Fig. 3C). We determined the difference between mean values in impacts on helmets with and without the add-on liner. Since the impacts were destructive, three impacts were performed for each condition. We also performed the t-test to determine the significance of the differences (p value). The largest reduction was observed for the frontal impact with a 4.35 m/s speed, with 27% reduction in mean translational acceleration (p = 0.017) and 22% reduction in mean rotational acceleration (p = 0.03). This was followed by the frontal impact at 7.5 m/s with 19% (p = 0.018) and 18% (p = 0.038) reduction and side impact at 7.5 m/s with 17% (p = 0.12) and 13% (p > 0.2) reduction in mean translational and rotational accelerations respectively. However, the reductions in side and rear impacts were of borderline and no significance, respectively.

The effect of the impact site and velocity on both translational and rotational accelerations was significant, but translational and rotational accelerations did not follow the same trend. The frontal impact at 7.5 m/s produced the largest peak translational acceleration (73 ± 3 g without components and 59 ± 6 g with components), followed by the 7.5 m/s rear impact (61.1 ± 0.0 g without components and 58.9 ± 0.1 g with components) and the 7.5 m/s side impacts (50.8 ± 0.1 g without components and 42.4 ± 0.1 g with components). The largest peak rotational accelerations were produced by the 7.5 m/s frontal impact (8.5 ± 0.4 krad/s^2^ without components and 7.0 ± 0.1 krad/s^2^ with components), followed by the 7.5 m/s side impact (7.0 ± 0.2 krad/s^2^ without components and 6.1 ± 0.1 krad/s^2^ with components) and the 7.5 m/s rear impact (4.7 ± 0.1 krad/s^2^ without components and 4.6 ± 0.2 krad/s^2^ with components). As can be seen, the 7.5 m/s frontal impacts consistently cause the largest peak accelerations.

### The effect of viscoelastic components on brain strain

**Figure 4:**
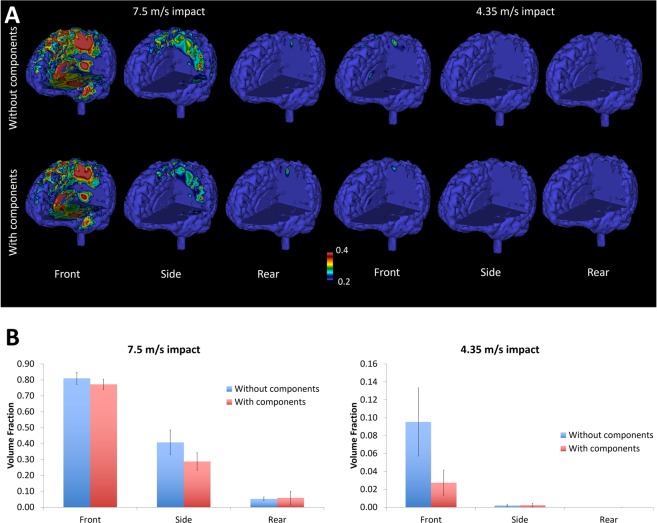
Computational results; (**A**) comparison of the strain contours (0.2–0.4) for the different impacts sites and velocities for specimens with and without the viscoelastic components; (**B**) comparison between cases with and without the viscoelastic component, of brain volume fraction averages exceeding 0.2 strain.

We used our computational model of traumatic brain injury to simulate the impacts and predict the maximum strain and strain rate at each finite element of the brain during the first 30 ms of the impacts. Figure 4(A) shows strain distribution across the brain for different impact velocities and directions. It can be seen that a large area of the brain sustains large strains in the 7.5 m/s frontal impact. This is followed by the side impact at 7.5 m/s. Other impacts do not produce large strains. Adding the viscoelastic components to the helmet has decreased strains in both frontal and side impacts at 7.5 m/s, with strain reduction in different areas including corpus callosum and sulci. Interestingly in contrast to the front impact, the side impact did not produce large strains in corpus callosum.

To better quantify the effects of including the viscoelastic components on brain strain, we evaluated the volume fraction of the brain that exceeded a 0.2 strain threshold, corresponding to onset of mild TBI^22,23^ (Fig. 4B). As expected the frontal impacts at 7.5 m/s affects a large volume of the brain (0.81 ± 0.04 without components and 0.77 ± 0.03 with components), followed by the side impacts at 7.5 m/s (0.41 ± 0.08 without components and 0.29 ± 0.05 with components). The enhanced helmet design decreased the volume undergoing large strains by 5% and 29% in the 7.5 m/s frontal and side impacts respectively but this reduction was not significant for the front impact (p = 0.29) and of borderline significance for the side impact (p = 0.11). Adding the liner seems to have reduced the strain in the frontal impacts at 4.35 m/s and this reduction was significant (p = 0.04), but it slightly increased the strain in rear impact at 7.5 m/s. In these impacts the volume fractions of the brain exceeding the strain threshold were small.

### The effects of viscoelastic components on brain strain rate

**Figure 5:**
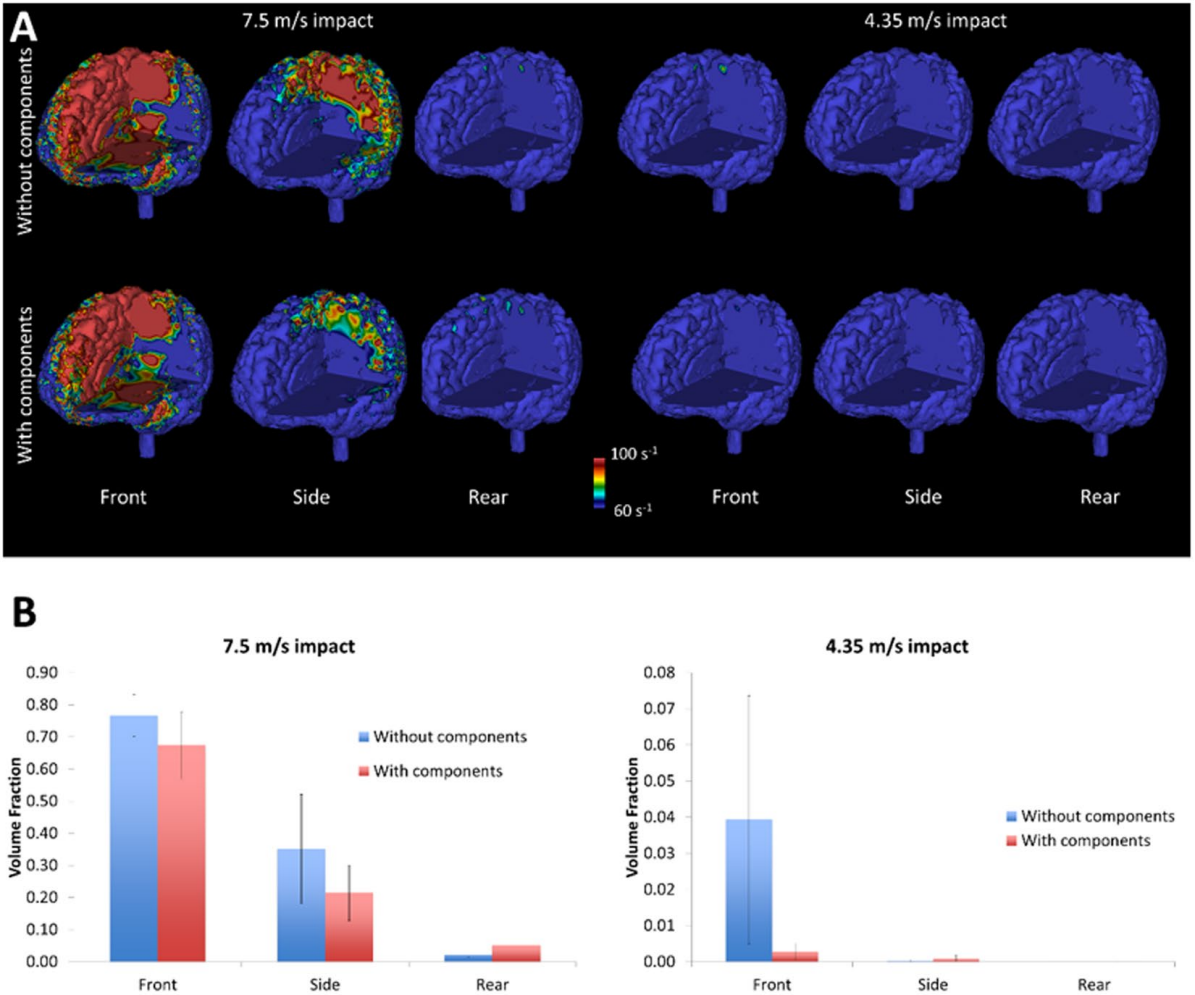
Computational results; (**A**) strain rate contours for the tested impacts showing the protection effect of the viscoelastic components; (**B**) brain volume fraction of elements that have reached at least 60 s^−1^ strain rate during deformation.

Figure 5(A) shows the distribution of strain rate across the brain for all impacts. A comparison between this figure and Fig. 4(A) shows that strain and strain rate have similar distributions, indicating that areas that undergo large strains also sustain high strain rates. The frontal impact at 7.5 m/s produced the largest strain rates followed by the side impact at 7.5 m/s. Adding the viscoelastic components to the liner reduced strain rate in both cases. Other impacts did not produce significant strain rates.

To better quantify the influence of using the viscoelastic components on strain rate, we evaluated the volume fraction of the brain tissue that exceeded a 60 s^−1^ strain rate, which is thought to be the threshold for mild TBI^22,23^. Figure 5(B) indicates that a large volume of the brain was under large strain rates in the frontal impact at 7.5 m/s (0.77 ± 0.06 without components and 0.67 ± 0.10 with components) followed by the side impact at 7.5 m/s (0.35 ± 0.17 without components and 0.21 ± 0.09 with components). The viscoelastic components reduced the strain rate for these impacts by 12% and 39% respectively, but the reductions were not statistically significant (p > 0.2). The new helmet design increased the strain rate for the rear impact at 7.5 m/s, but the volume fraction of the brain exceeding the threshold was not large (0.02 ± 0.01 without components and 0.05 ± 0.06 with components).

## Discussion

Our results show that adding the dilatant viscoelastic components on the interior surface of the liner of a high-performance helmet can reduce peak head accelerations as well as large strains and strain rates across the brain during oblique impacts. Our helmet test set-up allowed us to test helmets under oblique impacts, which are the dominant type of impacts in real-world accidents^24^, and to measure translational and rotational accelerations of the headform, which are commonly used to assess the performance of helmets. We extended this approach by using a computational model of traumatic brain injury to predict contours of maximum strain and strain rate across the brain. This enabled us to better assess the effects of adding the components to the helmet.

The improvements in the performance of the helmet with the add-on liner are due to the combined effects of the geometry and material of the components. The design of the viscoelastic components consisted of two interacting parts with a circular shape. The circular shape gives a transversely isotropic property to the components, meaning that their shearing properties tangential to the head do not depend on the direction of the head/helmet relative motion. In addition, the shape of the parts and their interaction allows for multimodal deformations, with different response in compression and shear. In addition to the geometry, the polymer material^25^ allows for energy dissipation at the nanoscale through molecular interactions^26^. The material is soft when deformed slowly, which makes it suitable for wearables and also for producing components with complex geometries through the injection moulding process as it can be easily removed from the mould. At high rates of deformation, the material stiffens to increase energy dissipation during impacts. This behaviour can be observed in shear relaxation modulus curve of the material, shown in Fig. 1A. The ratio between the short-term and long-term shear moduli of Rheon is nearly 15. The figure (Fig. 1A) also shows the relaxation modulus of two typical rubbers that are used in anthropometric test devices, among other applications. Butyl rubber has a comparable short-term stiffness but it is quite stiff in long-term response. Vinyl on the other hand is as soft as Rheon in long term but its short term stiffness is very low. EPS however does not show viscoelastic behaviour in 500 ms duration of the loading. The energy absorption mechanism of the EPS is through crushing of the microstructure and different to the elastomers. It is a stiff material, which makes it uncomfortable and necessitates the use of comfort padding in helmets. Amongst the materials discussed, Rheon shows the desirable characteristics for an add-on internal liner.

The components used in this study were 4.5 grams each, adding a total mass of 54 grams to the helmet, i.e. only 4% increase in the 1.31 kg mass of the helmet. Weight is an important design constraint and lightweight viscoelastic components could provide with promising solutions for improving protection capacity of helmets without significant burden on the mass. Additionally, such a small increase in mass will have a negligible effect on the head acceleration. Previous work has shown that an increase of 20% in the mass of the head/helmet assembly can decrease the head acceleration by only 10% ^27,28^. This was further confirmed with a one-dimensional model of helmeted head impact, which showed that there is an inverse relationship between peak head acceleration and square root of mass, suggesting that a 4% increase in mass will decrease the head acceleration by 2%. However, the acceleration reductions obtained with the add-on viscoelastic components were significantly larger than this value and varied with site of impact. Hence we conclude that, although the added mass will have an effect, the acceleration reductions were mainly driven by the geometry and material properties of the add-on components, rather than the increased mass.

Helmet performance and improvement varied with the site of impact. The frontal impacts at 7.5 m/s produced the largest head peak accelerations and brain strain and strain rate, followed by side impacts at 7.5 m/s. Including the viscoelastic components in the helmet reduced these injury metrics for both frontal and side impacts at 7.5 m/s, with largest reduction for the side impacts. The directionality in helmet performance is a consequence of the biomechanical properties of the head and neck as well as the helmet design. Here we used a Hybrid III neck, which has different behaviour during extension, flexion and lateral bending, similar to the human neck. On the other hand, helmets have different shapes and properties at different sites. The relatively wide cut-out of the shell at the front site, which is necessary to provide appropriate field of vision, makes this site of the helmet weaker than back, side and crown areas. This partly explains the measured higher peak accelerations and simulated brain strain and strain in the frontal impacts. At the back of the helmet, the foam liner is very thick (approximately 4 cm compared to 2 cm at the front) and the shell has a pronounced edge, allowing for more deformation, thus higher energy absorption and lower head accelerations and strains and strain rates. The presence of the edge near the impact location also means that a small difference in impact location would cause large variations in accelerations, as can be seen in Fig. 3A. Our results show that the highest injury would occur in frontal impacts and then side impacts, and interestingly the viscoelastic components were most effective in these impacts. The relatively limited number of specimens did not allow for extensive statistical analysis, but the results show promise in using the add-on viscoelastic components. Future work should focus on modelling the head, helmet and add-on liner system in order to distinguish and quantify the contribution of each element in head protection. Reliable models would allow for optimising the placement and geometry of the viscoelastic components to improve performance, particularly in frontal impacts.

The effect of adding the components for the front impact was comparable to the effect of the EPS enhancement of the helmet design in the back (in terms of translational accelerations) and the EPS enhancement of the helmet on the side (in terms of rotational accelerations). Adding just a few components in the front seems to have had the same effect in reducing translational accelerations as the design enhancement of the back site of the helmet with EPS foam (for 7.5 m/s). Similarly the added components reduced the rotational accelerations on the front impact to the levels of the enhanced EPS foam on the side of the helmet (for 7.5 m/s). This shows promise in the performance of the add-on components. Future work can involve optimising the geometry of the components and testing other promising materials in order to design more effective and lighter helmet designs.

We measured head accelerations during impacts. Peak head accelerations are suggested as physical parameters that correlate with risk of brain injury^13–15,23,29^, and peak translational acceleration is currently used in standard test methods to assess the protection capability of helmets^9–12^. Reconstructing 21 motorcycle accidents, the translational and rotational acceleration thresholds for AIS 2 head injuries was found to be 150 g and 8 krad/s^2^ ^30,31^. The head accelerations in our experiments were below these values, except for frontal impacts at 7.5 m/s on helmets without the viscoelastic components. Adding the viscoelastic components reduced head accelerations to a value lower than these thresholds.

The results suggest that there is discrepancy between the helmet assessment outcomes using translational acceleration, rotational acceleration (Fig. 3B) and mechanical metrics predicted by using the TBI finite element model (Figs 4B and 5B). Both translational and rotational head accelerations describe the global kinematics of the head, ignoring its complex structure including brain, meninges and cerebrospinal fluid. A better approach is to predict related biomechanical factors within brain tissue, such as stress, strain and strain rate, which have been correlated with damage to nerve cells and blood vessels^19–21,32^. Detailed computational models of TBI have allowed researchers to predict such factors in real-world impacts. Zhang reconstructed incidents based on NFL accidents and found that the shear stresses in the brain stem can be a strong predictor of mild TBI^23^. Ghajari *et al*.^18^, used a finite element model of TBI incorporating detailed anatomy of the brain, e.g. sulci, to reconstruct an NFL collision, a motorcycle accident and a fall. They found that maximal strains and strain rates are focused in sulcal regions, the same regions where pathology of the neurodegenerative disease chronic traumatic encephalopathy (CTE) has been observed. The same computational model was used in this study to evaluate brain tissue deformation and the resulting strains and strain rates during impacts, in order to better assess the performance of the helmets with and without the viscoelastic components.

We used strain and strain rate, as they are likely mechanical factors that damage nerve cells and blood vessels, with acute and long-term effects. Strain and strain rate have been studied using far field strain characterisation of neurons embedded in matrix. Strain and strain rates were found to have significant and distinctive effect on the injury pathology^33^. Arguably the scale of the studied matrix in^33^ is comparable to the element size used in our model and therefore the study of resulting strains and strain rates within the brain model could be of relevance in qualitatively assessing the potential neural and blood vessel damage and subsequently the effectiveness of helmet liners. A combination of experimental and numerical tools would be needed in developing protective strategies and systems for protecting sensitive regions of the brain that have been linked to injury.

This study is limited by the small number of specimens of a single material and geometry used. Helmet cost and the destructive nature of the tests were restrictive factors in the number of tests. This study therefore may be regarded as preliminary due to the relatively small number of samples. More tests would be required to establish more powerful statistical significance.

Overall, the results suggest that retrofitting the lightweight viscoelastic components into the helmet can have a measurable effect on TBI mitigation, which is attributed to the combined effects of the geometry and material of the components. Further experimental and computational work is needed to fully explore the potential of similar materials in mitigating TBI and to optimise the geometry and layout of the components to improve their performance. The combination of experiments and TBI modelling would allow for more comprehensive studies in the development of strategies that can potentially target the protection of specific regions of the brain that are affected during TBI.

## Materials and Methods

### Materials

The viscoelastic components were made of an active silicone^26^, with the trade name Rheon. Reacting polydimethylsiloxane with boron creates polyborodimethylsioxane, which has several hydrogen bonds. The hydrogen bonds can move slowly along the polymer chain at low rates of deformation but they show resistance to high deformation rates, producing the stiffening effect of the material^34^, as demonstrated by the shear relaxation modulus shown in Fig. 1A. The material is injection mouldable. The viscoelastic components were manufactured by injecting the material into a 3D printed mould, made through direct metal laser sintering process. The viscoelastic components were glued on the interior surface of the liner of a high-end motorcycle helmet, GP-Tech produced and provided by Dainese SpA (Fig. 2A).

### Impact experiments

We performed oblique impacts on the helmets using a new test rig^35^. The apparatus features an instrumented Hybrid III dummy head/neck system suspended on a pendulum to mimic the effects of the body in real-world crash scenarios. The axis of rotation of the pendulum was 7 cm posterior and 1.8 m superior to the lower neck. The mass of the pendulum was 48 kg and its moment of inertia about the axis of rotation was 58 kgm^2^. Using the distance from the lower neck to the axis of rotation (L) and the moment of inertia about this axis (I), the effective mass of the pendulum would be (I/L2 = ) 18 kg, which is close to the 16 kg mass that was used in previous work^36^ to represent the effects of the torso. A 13 kg oblique metal anvil was fitted on a drop tower with two guide bars (Fig. 2B). The anvil was covered with sand paper (grade 80) that was changed regularly. The impact was delivered by releasing the anvil from a certain height to reach a desirable impact velocity. The velocity of the anvil was measured by interrupting a light beam placed at a distance from the impact point, which showed less than 3% difference between the actual and intended impact velocities. The Hybrid III head and neck was calibrated by the manufacture and after every few tests, we checked the torque on the lower neck bolt to ensure consistency in neck tension. The helmets were impacted on the front, side and back at 4.35 m/s and 7.4 m/s impact velocities. Each test was recorded using high-speed photography. The headform was instrumented with a nine accelerometers array to measure six degrees of freedom kinematics of the head, i.e. translational and rotational acceleration vectors, during the impact (Fig. 2C). We used PCB 352C23 single axis miniature accelerometers, which are 0.2 grams and have a sensitivity of 5 mV/g and measurement range of ±1000 g. We logged the acceleration data at a 20 kHz sampling frequency using a rack of NI 9234 modules and an in-house LabView program. The details of the array and the relationship between linear accelerations measured with the array and rotational accelerations of the headform are explained in^37,38^. The recorded translational and rotational accelerations were then filtered with a 4^th^ order Butterworth filter at an 800 Hz cut-off frequency, and then used to load the computational model of TBI (Fig. 2D).

### Computational modelling of TBI

A three-dimensional finite element (FE) model of traumatic brain injury was used^18^ to predict strain and strain rate across the brain during the impacts (Fig. 2E). The model was developed by using high-resolution magnetic resonance (MR) images of a healthy 34 year-old male subject. The images were segmented and then an in-house code was used to generate the mesh. The FE model has nearly one million hexahedral elements and a quarter of a million quadrilateral elements representing different tissues, including the scalp, skull, brain, meninges, subarachnoid space and ventricles, as well as anatomical features such as sulci. The nonlinear and rate dependent mechanical behaviour of these tissues were modelled by using a hyper-viscoelastic material model^18^. The model combines a second degree Ogden model for the nonlinear response with a convolution integral for the viscoelastic response^18^. Material constants of the model, reported in^18^, were determined by fitting the model to results of quasi-static and dynamic tests on human brain tissue^18,39,40^. Translational and rotational accelerations were measured during the impact experiments to load the model. The highly nonlinear equations were solved with the ls-dyna code^41^. An in-house code was used to determine the maximum principal Green-Lagrange strain for each finite element of the brain using its nodal displacement and velocity vectors and to write the maximum value of this quantity across the loading duration in the vtk and nifti formats.

## Data Availability

The experimental datasets and numerical results generated during the current study are available from the corresponding author on reasonable request.
